# Age, race, and education as moderators of post-stroke cognitive decline following dental care

**DOI:** 10.3389/fstro.2026.1807730

**Published:** 2026-07-07

**Authors:** Michael H. Parrish, Leonardo Bonilha, Karly Pikel, Caitlin Scott, Stefanie Wood, Haley N. VerKuilen, Souvik Sen

**Affiliations:** 1 Department of Neurology, University of South Carolina School of Medicine, Columbia, SC, United States; 2 Division of Neurology, Prisma Health Richland Hospital, Columbia, SC, United States

**Keywords:** aging, cognitive decline, dental care, oral health, stroke

## Abstract

Post-stroke cognitive decline (PSCD) poses a significant challenge to long-term recovery and quality of life following stroke, influenced by both fixed biological factors and modifiable health behaviors such as oral and dental care. In this data-driven exploratory analysis of the PREMIERS Phase II randomized trial (ClinicalTrials.gov NCT#02541032), we examined the moderating effects of clinical, biological, and demographic characteristics on the relationship between dental care and PSCD over a 12-month period. The study included 280 stroke/transient ischemic attack (TIA) survivors who received either intensive or standard dental care. Cognitive outcomes were assessed using the Montreal Cognitive Assessment (MoCA) at baseline and follow-up, with change in MoCA score as the primary outcome. Lasso regression was applied for empirically based feature selection of moderators, and bootstrapped multiple linear regression demonstrated that increased dental visits predicted relatively better cognitive outcomes in older adults (age interaction-term *b* = −0.664, *p* < 0.001), Black participants (race interaction-term *b* = −0.475, *p* < 0.05), and those with low-intermediate education levels (education interaction-term *b* = 0.413, *p* < 0.05). Exploratory graphs revealed that older adults, Black adults, and adults with low-intermediate education showed greater cognitive improvement with higher dental visit frequency, with the final model (including selected moderators) significantly predicting PSCD [*F*_(11, 268)_ = 10.51, *p* < 0.001]. These findings highlight the potential of equity-focused, precision-medicine interventions that incorporate dental care to mitigate PSCD in vulnerable stroke populations.

## Introduction

Post-stroke cognitive impairment (PSCI) and post-stroke cognitive decline (PSCD) represent major challenges to long-term recovery and quality of life following stroke. PSCI refers to cognitive deficits detected at a single post-stroke timepoint, while PSCD captures change in cognitive performance across multiple assessments. Both outcomes are clinically consequential: PSCI and PSCD—including progression to dementia—are associated with functional independence and quality of life (Quinn et al., 2018; El Husseini et al., 2023; Sadigh and Volovici, 2024), stroke recurrence (Moroney et al., 1997), and increased risk of mortality (Melkas et al., 2009; Oksala et al., 2009).

The cognitive consequences of stroke are shaped by complex interactions between biological injury and individual characteristics. Structural and vascular features such as lesion location, stroke subtype, and infarct volume contribute significantly to outcomes (Aam et al., 2020; Aamodt et al., 2023; Levine et al., 2025). For example, left hemisphere and posterior circulation strokes are particularly associated with cognitive decline (Pendlebury and Rothwell, 2009; Siow et al., 2024), and medial temporal atrophy has differential effects depending on stroke laterality (Aamodt et al., 2022). These injury-related factors are typically fixed and not easily modifiable. However, demographic and clinical variables—such as age, sex, education, and comorbidities—also influence post-stroke cognitive trajectories and may provide avenues for targeted intervention (Rost et al., 2022; El Husseini et al., 2023).

In recent years, modifiable health behaviors and lifestyle factors have garnered attention for their potential to mitigate PSCI and PSCD. Physical activity, cognitive stimulation, sleep quality, and nutrition are increasingly studied for their protective roles (Obaid et al., 2020; Huang et al., 2022). Aerobic exercise, in particular, has shown benefit for cognitive performance in stroke survivors (Moore et al., 2015), while treatment of sleep disorders like obstructive sleep apnea may also preserve cognition (Aaronson et al., 2016; Zhang et al., 2023). These insights have supported the development of multifactorial interventions, though effect sizes remain modest (Sun et al., 2021).

One emerging, yet underexplored, area for intervention is oral and dental health. Poor periodontal status may contribute to post-stroke cognitive decline through systemic inflammation, microbiome dysregulation, and vascular dysfunction (Zhang et al., 2020; Wei et al., 2023; Shiraishi et al., 2024). Oral health is closely tied to immune aging (Ebersole et al., 2018), and may influence cognition through processes such as “inflammaging”—chronic, low-grade inflammation linked to aging and neurodegeneration (Finger et al., 2022). Modifying oral inflammation and health may thus offer a unique, scalable pathway to improve cognitive resilience, particularly in older adults.

We recently explored this possibility in the Periodontal Disease Treatment After Stroke or Transient Ischemic Attack (PREMIERS) Phase II randomized trial, which demonstrated that intensive periodontal treatment was safe and effective in improving vascular outcomes such as blood pressure and HDL levels (Sen et al., 2023). In the present study, we conducted a secondary analysis of PREMIERS to evaluate changes in cognition over a 1-year period, with a focus on identifying subgroups most likely to benefit from oral health care.

This analysis prioritized change-based assessment of PSCD, which more accurately captures stroke-related cognitive shifts than cross-sectional measures that may reflect preexisting pathology (El Husseini et al., 2023). Using a data-driven approach, we examined whether variables such as age, race, education, and sex moderated the relationship between dental care and PSCD. Understanding which populations benefit most could inform precision-based strategies for preventing cognitive decline in stroke survivors.

## Materials and methods

### Study population and design

The PREMIERS clinical trial methods and results have been reported elsewhere (Sen et al., 2023; clinicaltrials.gov; Unique identifier: NCT 02541032), but the study will be summarized briefly here (Pikel et al., 2025). PREMIERS was a multicenter phase II trial for which the primary objective was to evaluate the effect of intensive periodontal treatment on recurrent vascular events among stroke and high-risk TIA survivors. The sample of 280 patients was randomized in a 1:1 ratio to either intensive (*n* = 140) or standard arm (*n* = 140). Study activities were conducted in the clinical settings of the Neurology and Dentistry departments at both University of South Carolina Prisma Health Richland and The University of North Carolina at Chapel Hill. The study was approved by the Institutional Review Boards of both institutions. The results of this paper are based on a secondary analysis of the original study outcome data. This paper focuses on an analysis of all participants irrespective of treatment assignment, specifically examining the effects of number of dental visits attended on cognitive decline after the ischemic event.

### Periodontal intervention

Intensive treatment involved supragingival and subgingival scaling and root planning using hand instruments and ultrasonic scalers under local anesthesia, extraction of teeth evaluated as being nonrestorable or too severely affected by periodontitis, administration of local antibiotics, and provision of oral hygiene instructions. At baseline, intensive treatment participants were also given a Philips Sonicare ultrasonic toothbrush and an interdental cleaner with antibacterial mouth rinse. Standard treatment involved full-mouth supragingival scaling to remove only supragingival plaque and calculus and supragingival polishing with abrasive dental polishing paste. Standard treatment patients were also given education about their PD severity and referred to care if their oral condition needed immediate attention. Regardless of treatment assignment, all study participants were monitored for safety and progression of disease. At the end of the yearlong study period, the standard treatment group was offered the dental care involved in the intensive treatment.

### Cognitive assessment

The Montreal Cognitive Assessment (MoCA) was administered at baseline and at the end of the 12-month study period. MoCA is an effective screening tool for PSCI and has been used in multiple studies of cognitive decline (Tan et al., 2017; Siqueira et al., 2019). The assessment evaluates overall cognitive capacity out of a total score of 30. It is based on evaluations on seven cognitive domains: visuospatial/executive, naming, attention, language, abstraction, recall, and orientation (Shi et al., 2018). MoCA may be a more sensitive measure of vascular cognitive impairment changes (compared to the Mini Mental State Examination, for example) because it includes components which test frontal-executive function and abstract reasoning (Tan et al., 2017). For our study, the PSCD score was calculated by subtracting the baseline score from 12-month follow-up score. Research has validated the MoCA for PSCD through comparison and association with formal neuropsychological test battery changes (Tan et al., 2017).

### Baseline demographic, clinical, and brain health variables

Clinical and demographic information was obtained at the baseline visit before treatment began. Information was collected from the medical records about age/date of birth, sex, race, education level, hypertension, diabetes, substance use (smoking and alcohol), history of coronary artery disease, myocardial infarction, angina, coronary artery bypass graft, and atrial fibrillation (AFib). Level of education was operationalized as low-intermediate (high school degree or equivalent or less), or high (completion of college degree or pursuing college-level education). Hypertension status was operationalized as a previous diagnosis of hypertension, irrespective of current medication treatment. Substance use was assessed through evaluation of current or past smoking cigarettes and current regular consumption of alcohol. AFib was operationalized as either past diagnosis or presentation on electrocardiogram (EKG) at admission. Medication adherence was assessed through the Morisky Medication Adherence Scale (MMAS-8) scale (Morisky and DiMatteo, 2011). Periodontal profile class staging was assessed to stratify risk for periodontal disease (Morelli et al., 2017). The 12-month pre- to post-intervention change in this staging score was calculated to derive a measure of periodontal disease change. We assessed a measure of brain health by examining cerebral small vessel disease with the use of axial FLAIR imaging and the three-point Fazekas scale rating of white matter hyperintensities (WMH; lesions). MRI scans were acquired with a Siemens 1.5 T MRI scanner (Symphony Tim B17; Siemens Healthcare, Erlangen, Germany) or GE 1.5 T MRI Scanner (Discovery 450; MXR Imaging, San Diego, California, USA). Periventricular and deep WMH scores were rated separately (0–3) and then combined with a sum (0–6). Periventricular WMH (PVWMH) scores were rated in the following manner: 0 = absent; 1 = caps or pencil-thin lining; 2 = smooth “halo”; 3 = irregular periventricular signaling extending into the deep white matter. Deep WMH (DWMH) were rated in the following manner: 0 = absent, 1 = punctate foci; 2 = beginning of confluence of foci; 3 = large confluent areas of foci. TOAST subtype (transient ischemic attack, large artery atherosclerosis, lacunar stroke, cardioembolic, cryptogenic/embolic stroke of undetermined source; Adams et al., 1993), stroke territory (left anterior circulation, right anterior circulation, posterior circulation, both/mixed), and NIH Stroke Scale score (for measurement of stroke severity) were also evaluated and defined by the team's vascular neurologist.

### Statistical analyses

Statistical analyses were performed in R (R Core Team, 2023) and SPSS Version 29 (IBM Corp, 2022). First, descriptive statistics were analyzed for the candidate moderator variables: age, sex, race, BMI, NIHSS, income, hypertension status, diabetes status, coronary artery disease/myocardial infarction/coronary artery bypass graft, AFib, stroke territory, TOAST subtype, TIA status, history of smoking, current alcohol drinking status, and Fazekas WMH ratings. We specifically analyzed the distributions to examine any major outliers and visualized correlation plots to examine associations that could influence collinearity. We then completed missing variable cases with the multiple imputation approach implemented in the R package MICE (Multiple Imputation by Chained Equations). MICE uses iterative predictive models to fill in empty values by estimating relationships between variables. The MICE package creates multiple datasets for the missing values; to create a final dataset, we averaged missing values imputation values. To create interaction terms for each of the candidate variables, we first calculated *z*-scores for each candidate variable and multiplied these by the *z*-score for the number of study dental visits (labeled “Dental Visits”).

Primary data analysis was conducted in two steps: feature selection and final model estimation. In sum, we conducted a Lasso regression to automatically select the most important interaction terms in a data-driven fashion and then created a simplified multiple linear regression model for the purposes of interpretability and final significance testing. Lasso is a machine learning regression technique which involves introducing a L1 regularization penalty to the optimization function (James et al., 2023). This ultimately shrinks unimportant features to have a regression coefficient of zero. The non-zero regression coefficient terms are then used as the selected features for further analysis. Given the high number of interaction variables (*n* = 17) and the drawbacks of alternatives such as stepwise linear regression (e.g., multiple testing issues), we decided to employ Lasso regression in our analyses. After feature selection, we conducted a final bootstrapped multiple linear regression analysis with the feature-selected interaction variables and their component terms (the components were input as predictors in the final model regardless of Lasso regression results). Ninety-five percent of confidence intervals generated from bootstrapping were then examined to evaluate final significance levels for all predictors of PSCD. For interpretation, we used bar chart graphs to evaluate the overall pattern of results producing significant interactions. Given the exploratory nature of the current research, we did not pursue further statistical comparison in mean levels of PSCD at specific levels of the interaction term component variables.

We conducted sensitivity analyses in which we included intervention group (intensive vs. standard) as a covariate in the final simplified multiple linear regression model and repeated similar analyses in the stratified samples (intensive and standard subsamples). We also conducted a sensitivity analysis examining the effect of a behavioral confound that may have complicated the relationship between dental care and cognitive decline, medication adherence, as measured by Morisky Medication Adherence Scale (MMAS-8) scale. Lastly, to address potential confounding issues related to biological stroke characteristics, we tested whether including stroke territory and TOAST type variables in the final model influenced moderation effects.

To begin to explore mechanistic changes from the dental intervention, we were interested in examining the relationship between number of visits and pre- to post-intervention changes in periodontal disease status by conducting a Spearman's non-parametric rank correlation. We also wanted to examine whether replacing the dental visits predictor with periodontal disease status change would result in similar moderation effects in the final simplified multiple linear regression model.

## Results

### Candidate moderator variables distributions and associations

**Figure 1:**
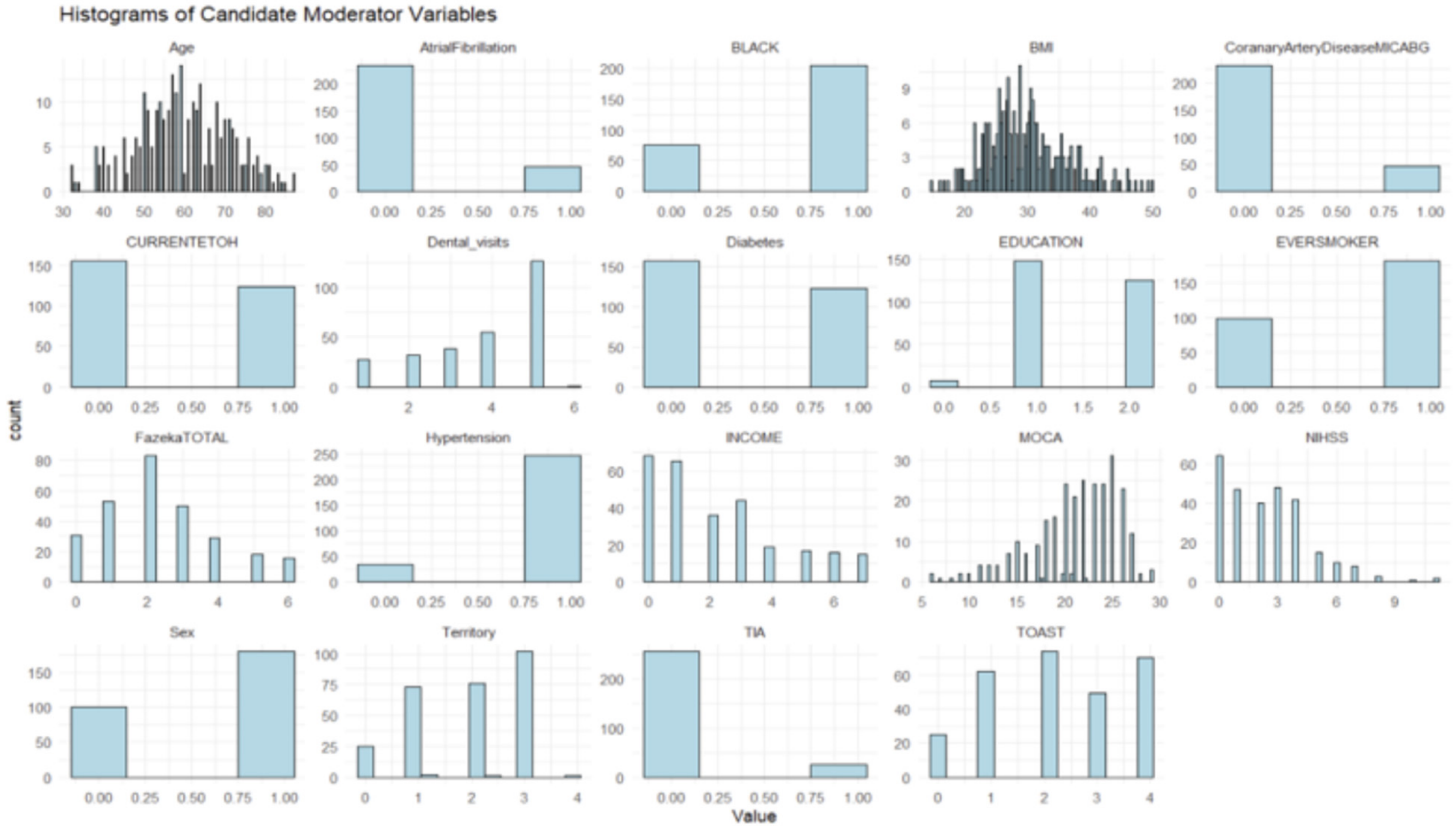
Histograms visualizing distributions of moderators tested in Lasso regression feature selection and multiple linear regression models.

Figure 1 shows the variable distributions for the candidate moderators for the effect of dental visits on PSCD. Notably, the distributions for dental visits, BMI, income, baseline MoCA, and NIHSS were skewed; however, given that final analyses were conducted with bootstrapping methods, we were not concerned with the potential impact on results in the final simplified model. Supplemental Figure 1 shows the correlation matrix for the candidate variables. No variables were strongly correlated; therefore, multicollinearity was not expected to be an issue for regression analyses. While specific quantitative analyses and null hypothesis significance testing were not applied to these pairwise correlations, the strongest associations were found between: TIA and territory, TIA and TOAST type, race (Black) and age, race (Black) and income, NIHSS and baseline MoCA, baseline MoCA and income.

### Feature selection results

Lasso regression analyses revealed that age-dental visits, race-dental visits, education-dental visits, and NIHSS-dental visits interaction variables were selected as significant features worth exploring in the context of multiple linear regression modeling. The other 13 variables had their interaction term regression coefficients shrunk to zero given a lack of substantial relationship with the outcome variable PSCD. The order of the regularized weight's magnitude/importance (descending order) was: race × dental visits (−0.216), age × dental visits (−0.214), education × dental visits (0.096), and NIHSS × dental visits (−0.008). In addition, sex (female) and baseline MoCA variables were also selected based on Lasso regression results. Simplified model results multiple linear regression (MLR) analyses were then run with the interaction variables selected from feature selection and their component terms (e.g., both age and dental visits variables would be included because their interaction term was selected). The following variables were included: dental visits (count), sex (female), race (Black), baseline MoCA, age, education, NIHSS, age × dental visits, race × dental visits, education × dental visits, and NIHSS × dental visits. The full simplified model significantly predicted PSCD [*F*_(11, 268)_ = 10.51, *p* = 5.17 × 10^−16^]. The predictors explained a significant amount of variance in the outcome: multiple *R*^2^ = 0.301, adjusted *R*^2^ = 0.273. The following were found to be significant predictors in the simplified MLR model: dental visits × age (*b* = −0.664, *p* < 0.001; CI: [−1.067, −0.240]), dental visits × race (Black) (*b* = −0.475, *p* = 0.023; CI: [−0.903, −0.059]), and dental visits × education (*b* = 0.413, *p* = 0.043; CI: [0.035, 0.826]), sex (female) (*b* = −0.992, *p* = 0.018; CI: [−1.807, −0.170]), baseline MoCA (*b* = −0.439, *p* < 0.001; CI: [−0.570, −0.314]).

### Graphical exploration of age, race, and education interaction effects

**Figure 2:**
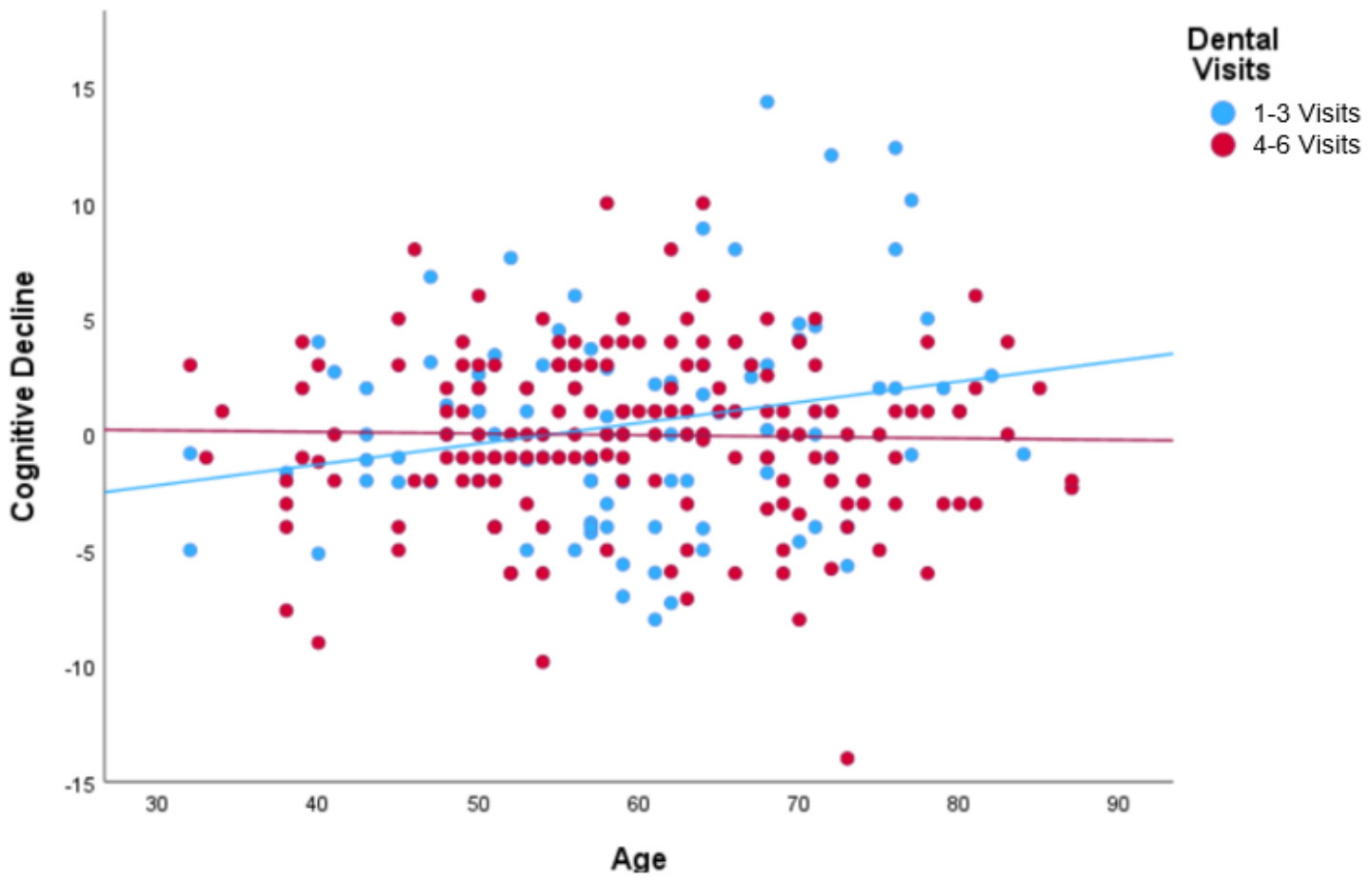
Scatterplot visualizing interaction between number of dental visits and age influencing post-stroke cognitive decline.

**Figure 3:**
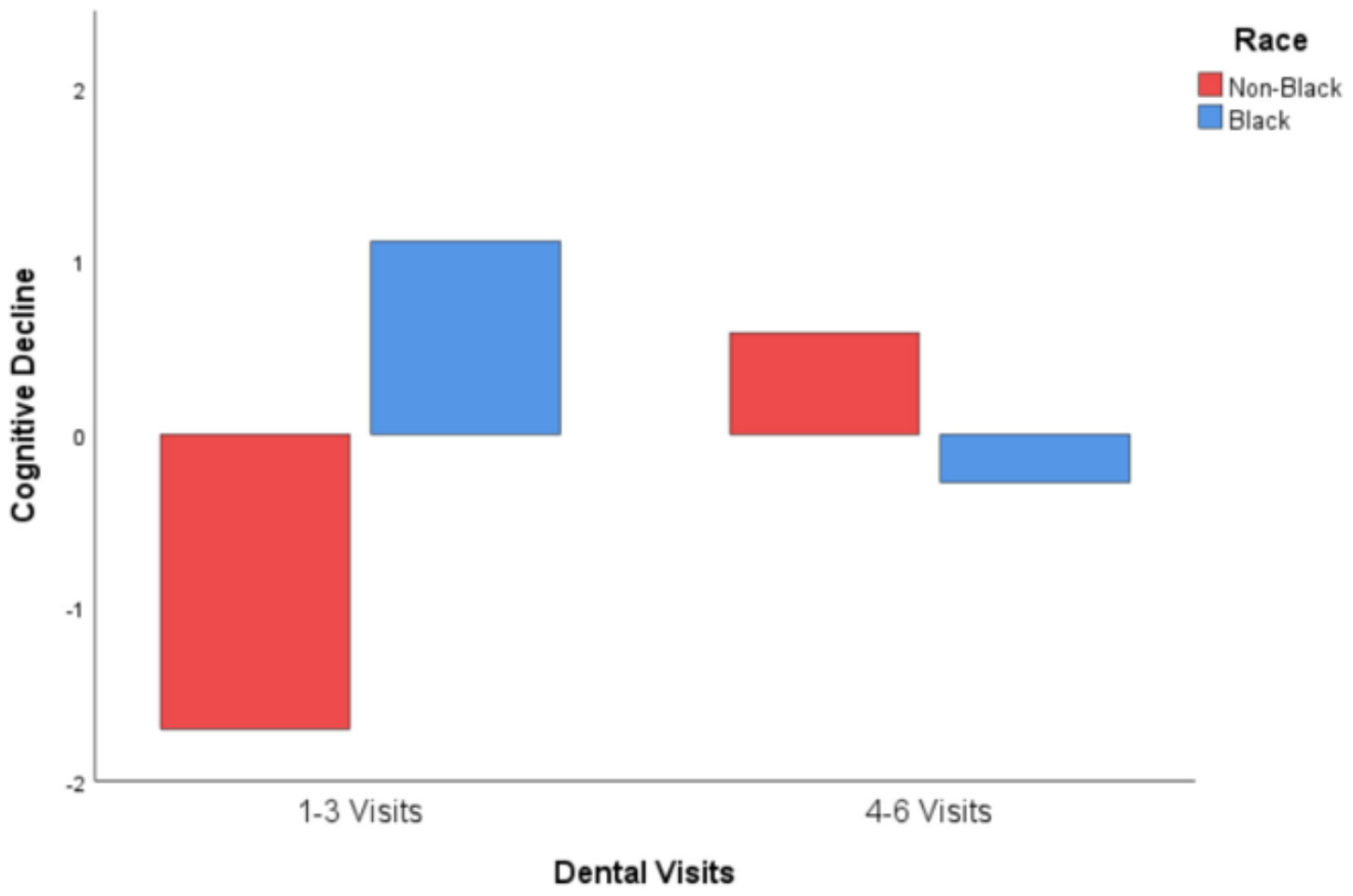
Bar chart visualizing interaction between number of dental visits and race influencing post-stroke cognitive decline.

**Figure 4:**
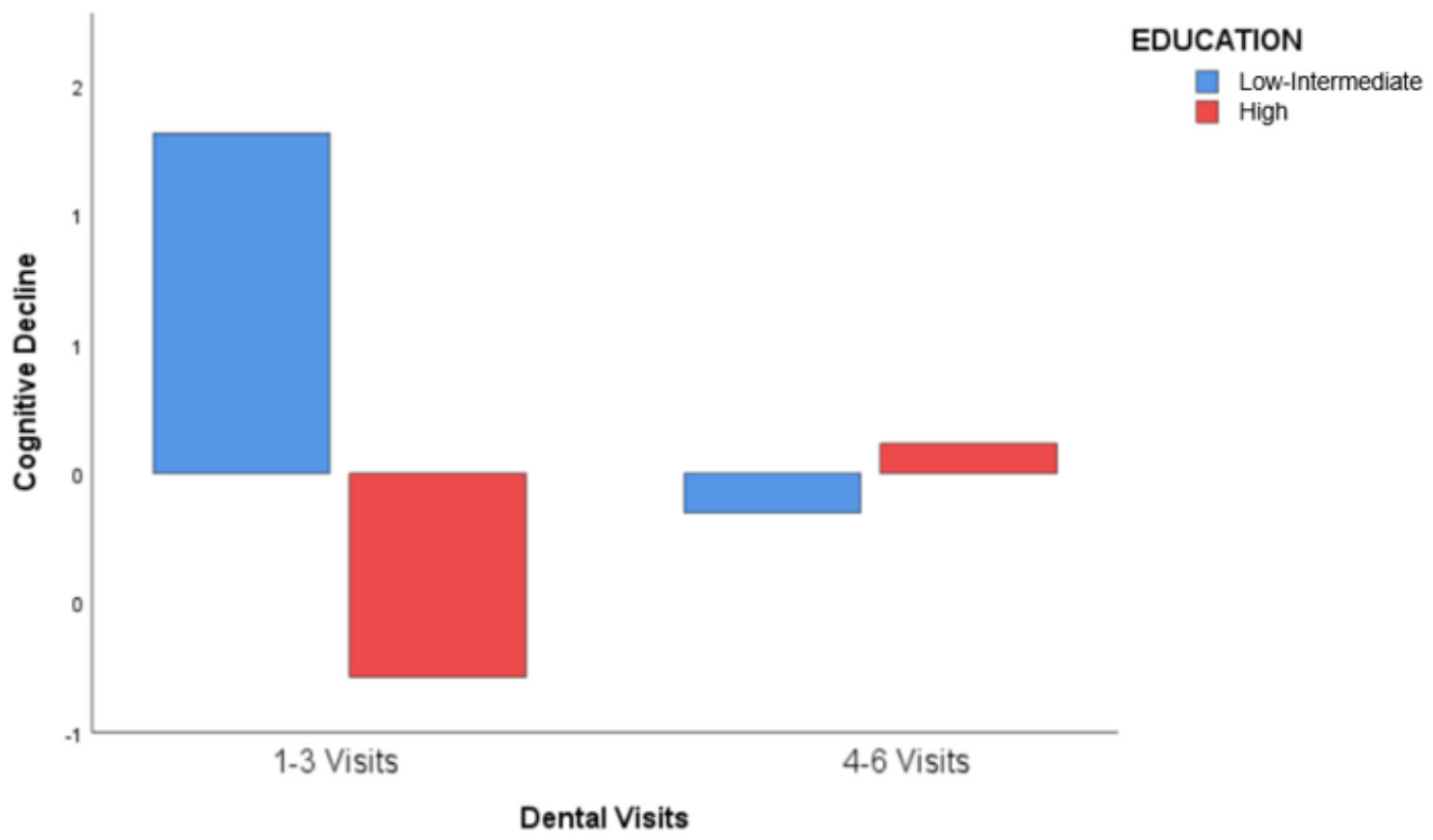
Bar chart visualizing interaction between number of dental visits and education influencing post-stroke cognitive decline.

Figures 2–4 depict exploratory visualizations of the relationship between significant moderators, number of dental visits, and PSCD. Following statistical analysis, we next interrogated the three significant interaction effects by visually inspecting the specific relationships between different levels of the moderators and predictors and the outcome variable PSCD. For the scatter plot depicting the significant dental visit × age interaction, visual inspection revealed that increasing number of dental visits was uniquely beneficial for older adults, specifically those in their seventies. This benefit was not similarly apparent for adults younger than 70 years old. For the bar graph depicting the significant dental visit × race (Black) interaction, we found that higher number of dental visits was uniquely beneficial for Black adults. Surprisingly, non-Black participants showed increased levels of cognitive impairment at the highest numbers of dental visits. For the bar graph depicting the significant dental visit × education interaction, we found that only adults with low-intermediate levels of education (high school graduate, no college degree or higher) benefitted from the increased number of dental visits. However, it should be noted only seven participants reported basic level of education. In summary, the post-stroke cognition of older adults, Black adults, and adults with low-intermediate levels of education appeared to benefit most from increasing effects of more dental treatment.

### Sensitivity analyses: examining the effects of intervention group, medication adherence, and stroke characteristics

When including the intervention group control variable into the overall simplified multiple linear regression model, we observed slight changes to the model results but the general pattern remained consistent. The following were found to be significant or marginally significant predictors in this updated MLR model: dental visits × age (*b* = −0.656, *p* = 0.004; CI: [−1.094, −0.200]), dental visits × race (Black) (*b* = −0.482, *p* = 0.038; CI: [−1.002, −0.042]), dental visits × education (*b* = 0.406, *p* = 0.039; CI: [0.046, 0.813]), sex (female) (*b* = −0.997, *p* = 0.027; CI: [−1.921, −0.197]), baseline MoCA (*b* = −0.441, *p* < 0.001; CI: [−0.563, −0.309]). When examining the stratified analyses, results were more consistent with the original overall pattern for the intensive treatment subsample. However, the analysis of the standard treatment group revealed the only significant moderation effect for dental visits × age at standard thresholding. On the other hand, the analysis of the intensive treatment group revealed the only significant moderation effect for dental visits × education. The following were found to be significant or marginally significant predictors in standard treatment subsample MLR model: dental visits × age (*b* = −0.624, *p* = 0.029; CI: [−1.313, −0.137]), sex (female) (*b* = −1.294, *p* = 0.062; CI: [−2.696, −0.002]), age (*b* = −0.052, *p* = 0.096; CI: [−0.118, 0.010]). The following were found to be significant or marginally significant predictors in intensive treatment subsample MLR model: dental visits × age (*b* = −0.654, *p* = 0.060; CI: [−1.265, 0.096]), dental visits × race (Black; *b* = −0.605, *p* = 0.066; CI: [−1.264, 0.036]), dental visits × education (*b* = 0.688, *p* = 0.025; CI: [0.085, 1.247]), baseline MoCA (*b* = −0.394, *p* < 0.001; CI: [−0.560, −0.232]).

When examining the effect of the potential behavioral confound medication adherence, we found overall similar results to our main model findings. The following were found to be significant or marginally significant predictors in this updated MLR model: dental visits × age (*b* = −0.687, *p* = 0.005; CI: [−1.102, −0.247]), dental visits × race (Black) (*b* = −0.473, *p* = 0.033; CI: [−0.945, −0.049]), dental visits × education (*b* = 0.400, *p* = 0.052; CI: [0.016, 0.831]), sex (female; *b* = −1.008, *p* = 0.011; CI: [−1.779, −0.164]), baseline MoCA (*b* = −0.437, *p* < 0.001; CI: [−0.565, −0.314]).

Next, we aimed to examine the potential confounding effects of biological stroke characteristics in our sensitivity analyses. Including stroke territory and TOAST type did not majorly overall model results. The following were found to be significant or marginally significant predictors in this updated MLR model: dental visits × age (*b* = −0.656, *p* = 0.002; CI: [−1.077, −0.239]), dental visits × race (Black; *b* = −0.482, *p* = 0.025; CI: [−0.962, −0.100]), dental visits × education (*b* = 0.399, *p* = 0.067; CI: [0.007, 0.854]), sex (female) (*b* = −0.992, *p* = 0.017; CI: [−1.779, −0.166]), baseline MoCA (*b* = −0.439, *p* < 0.001; CI: [−0.559, −0.298]).

### Examining moderation effects based on periodontal disease status change

Correlational analyses examining the relationship between number of dental visits and periodontal disease status change revealed a highly significant negative association [*r*__*s*_(238)_ = −0.327, *p* < 0.001]. Thus, higher frequency of dental visits across both intervention conditions was associated with improvements in periodontal health outcome. Next, we input periodontal disease status change as the main predictor in the simplified MLR model, replacing number of dental visits and inputting interaction terms based on periodontal disease status change. We replicated a similar pattern of moderation effects for age and education, but we did not find a similar pattern when examining the interaction between periodontal disease status change and race. The following were found to be significant or marginally significant predictors in this updated MLR model: periodontal disease change × age (*b* = 0.491, *p* = 0.060; CI: [−0.106, 0.981]), periodontal disease change × education (*b* = −0.578, *p* = 0.025; CI: [−1.077, −0.012]), sex (female; *b* = −0.925, *p* = 0.042; CI: [−1.864, −0.095]), baseline MoCA (*b* = −0.361, *p* < 0.001; CI: [−0.488, −0.246]).

## Discussion

In this secondary analysis of the PREMIERS trial, we applied data-driven statistical approaches to identify three key demographic moderators—age, race, and education—that shaped the relationship between dental care and PSCD. Specifically, greater exposure to dental treatment was associated with improved cognitive outcomes among older adults, Black adults, and individuals with low to intermediate levels of education.

Age emerged as the most reliably significant moderator, with older individuals deriving the greatest cognitive benefit. This is consistent with the robust relationship between aging and vulnerability to PSCD (Lo et al., 2022). Age-related changes in brain structure and immune function—including diminished white matter integrity, vascular remodeling deficits, and altered inflammatory profiles—may predispose older adults to steeper cognitive decline (Cabeza et al., 2018; Rost et al., 2022; Aamodt et al., 2023). However, these same biological vulnerabilities may also enhance responsiveness to interventions that modulate systemic inflammation. Oral health interventions may help reverse components of “inflammaging” and restore immune resilience (Franceschi et al., 2018; Finger et al., 2022). While the current study did not adequately measure detailed periodontal parameters and oral inflammatory biology, it will be critical for future studies to delve into these factors to establish the mechanistic pathways between dental care, inflammation, aging, and cognitive outcomes. Aging is also linked to reduced functional connectivity in neural networks supporting psychological resilience (Chen et al., 2025), and it is plausible that health-promoting behaviors like oral care can strengthen these networks via improved self-efficacy and engagement. These mechanisms suggest that aging may not only be a risk factor but also a point of leverage for targeting cognitive outcomes in stroke survivors.

Race was also a significant moderator, with Black adults experiencing greater cognitive benefit from dental treatment. This is relevant to research showing that Black individuals often experience more severe PD, and greater susceptibility to PSCD (Levine et al., 2018; Sarfo et al., 2020; Gillone et al., 2023; Wang et al., 2023). This observed pattern may reflect underlying inflammatory burden, genetic predisposition, and differential intervention response. Despite these disparities, behavioral interventions—such as computerized cognitive training have shown greater positive effects in Black adults (Nwosu et al., 2024). Similarly, vascular interventions like hypertension management, offer a unique opportunity to mitigate racial cognitive disparities. For example, one cohort study found racial disparities in cognition between Black and White adults were in part explained by levels of systolic blood pressure (cognitive disparity was not statistically significant after controlling for systolic blood pressure; Levine et al., 2020). Given the systemic link between PD and vascular conditions such as hypertension, targeted dental interventions may yield the same effects. Our findings suggest that oral care may represent a similarly effective strategy for reducing cognitive disparities by targeting modifiable inflammatory and vascular pathways.

Perhaps most notably, educational attainment moderated cognitive outcomes in an unexpected direction: individuals with low-intermediate education, compared to high education (college degree) experienced the greatest benefit from dental care. This could appear contradictory with the assumption that higher education always confers greater cognitive resilience. Some evidence suggests that more highly educated individuals may harbor greater latent neuropathology that is masked by compensatory mechanisms until a threshold is passed—after which rapid cognitive decline ensues (Scarmeas et al., 2006; Verlinden et al., 2016). In stroke populations, executive function decline post-event has been found to be steeper in college-educated individuals (Springer et al., 2025). These individuals may also be less responsive to health interventions if their neuropathological burden is too severe to be no longer modifiable. By contrast, individuals with intermediate education may represent a “sweet spot” of cognitive reserve—where moderate pathology remains responsive to systemic interventions like oral care. This interpretation is consistent with findings from stroke cohorts in Asia and Europe, where lower education predicted worse recovery, but also suggested greater capacity for treatment-related gains (Shin et al., 2020; Elgh and Hu, 2023).

Additionally, it is noteworthy for future research focused on dental care intervention effects that we found baseline cognition and female sex were predictive of PSCD, however neither moderated the effect of dental visits. Higher baseline global cognition was related to lower levels of PSCD, potentially indicating the individuals with higher levels of cognitive reserve were more resistant to PSCD, although this has yet to be tested comprehensively (Tao et al., 2023; Li et al., 2022). Additionally, our results show that female sex, compared to male sex, related to decreased PSCD. While current overall research on sex-specific patterns of PSCD is mixed, this finding is in line with at least some evidence that women may be uniquely protected from neurological deficits in certain domains of cognition, such as verbal memory (Zinman et al., 2023; Exalto et al., 2023). Though these variables may not directly interact with dental care to influence cognitive health, they should be included in statistical models predicting PSCD to account for their independent effects.

Regarding our sensitivity analyses focused on examining the effect of intervention group, we found that inclusion of this group control variable did not majorly influence the general pattern of results. All three of the highlighted moderation effects were still statistically significant (age, race, and education). Related to our stratified analyses, results revealed that the overall pattern of these three moderation effects was only detectable in the intensive treatment condition. However, given the small to moderate sample size and fairly large number of predictor variables in the model, we advise caution when interpreting these subsample results. Similarly, the sensitivity analysis examining the potential confounding effects of medication adherence and biological stroke characteristics showed our results to be overall robust.

Concerning our analyses involving periodontal disease status change, we found complementary results which also begin to establish mechanistic linkages between dental intervention and biological mechanisms. Specifically, we found that the higher number of dental visits was associated with improvements in periodontal disease status. Also, we replicated a similar pattern of moderation effects for age and education when replacing number of dental visits with periodontal disease change. The cause of this specific inconsistency for the race moderation effect is potential avenue for future research.

Taken together, these findings suggest that interventions to prevent PSCD may be more effective when tailored to subgroups based on age, race, and educational background. Most existing cognitive rehabilitation strategies yield small to moderate effects (Oberlin et al., 2017; Sun et al., 2021), and few are designed with population-specific responsiveness in mind. Our results highlight the need for stratified trial designs, targeted recruitment strategies, and subgroup analyses to maximize and detect efficacy in vulnerable groups (Elkind et al., 2020; Chavez et al., 2024).

Despite the strengths of this study, several limitations should be acknowledged. The analysis was underpowered to detect small moderation effects, and findings related to race were essentially limited to comparisons between Black and White participants. Furthermore, all results were produced from a relatively small sample size and need to be interpreted with caution. Additionally, we did not measure depressive symptoms and depression may have confounded or mediated the statistical relationships between age, sex, education, dental care, and PSCD (Lo et al., 2022; Shin et al., 2022). Post-stroke depression is a prevalent condition and is known to influence longitudinal cognitive outcomes specifically (Butsing et al., 2024; Liang et al., 2023). Finally, cognitive outcomes were assessed only over 1 year; longer-term follow-up is needed to determine the durability of treatment effects.

In conclusion, this study identifies three subgroups—older adults, Black adults, and individuals with low-intermediate education—who may uniquely benefit from dental care as a strategy to mitigate PSCD. These findings support the integration of oral health into broader cognitive intervention frameworks and emphasize the need for equity-focused, precision approaches in stroke rehabilitation.

## Data Availability

The data analyzed in this study is subject to the following licenses/restrictions: None. Requests to access these datasets should be directed to “Data requests are considered by Principal Investigator SS (souvik.sen@uscmed.sc.edu).”

## Supplementary material

Supplemental Figure 1 Correlation matrix depicting bivariate relationships between candidate moderator variables.
