# Supplementary figures and images for: Age, race, and education as moderators of post-stroke cognitive decline following dental care
Source: Front Stroke. 2026 Jul 7;5:1807730. doi: 10.3389/fstro.2026.1807730 (PMC13386416; doi:10.3389/fstro.2026.1807730)

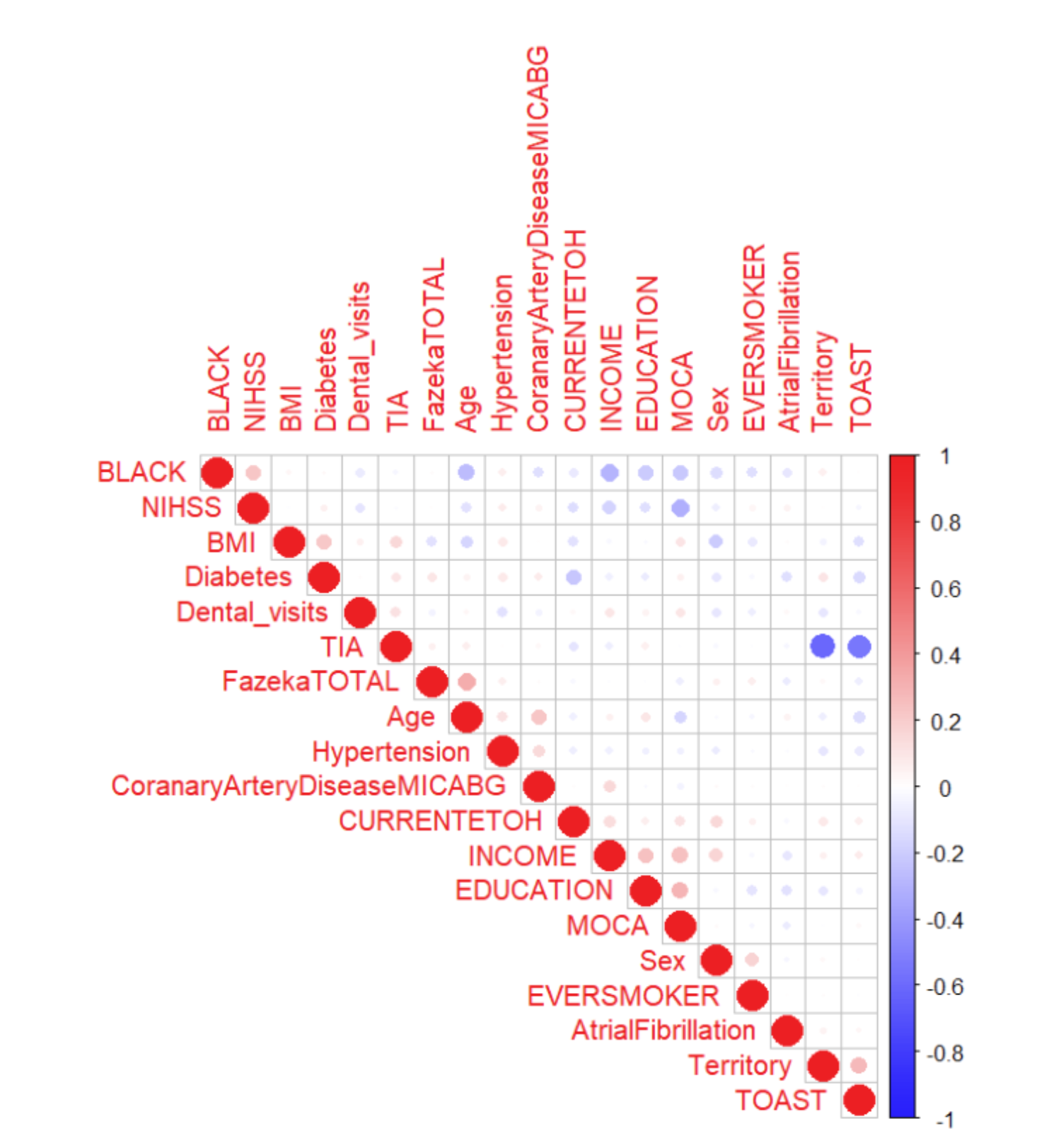

Supplement: Supplemental Figure 1 — Correlation matrix depicting bivariate relationships between candidate moderator variables. [file Image_1.tif]
